# miR-338-5p Targets Epidermal Growth Factor-Containing Fibulin-Like Extracellular Matrix Protein 1 to Inhibit the Growth and Invasion of Trophoblast Cells in Selective Intrauterine Growth Restriction

**DOI:** 10.1007/s43032-020-00160-3

**Published:** 2020-02-13

**Authors:** Hong Wen, Ying Hu, Lu Chen, Li Zhao, Xinyun Yang

**Affiliations:** Women’s Hospital, School of Medicine, Zhejiang University, Hangzhou, Zhejiang Province People’s Republic of China

**Keywords:** sIUGR, Trophoblast cell, miR-338-5p, EFEMP1, AKT

## Abstract

Selective intrauterine growth restriction (sIUGR) is a disorder of monochorionic (MC) twin pregnancies. However, the underlying mechanism remains largely unknown. Trophoblast cells are the major component of the placenta. Dysfunction of trophoblast cells is associated with placental dysfunction. Our previous study identified miR-338-5p is downregulated in placenta tissues sharing larger twins of sIUGR. In the present study, we aimed to investigate the role of miR-338-5p in trophoblast cells and explored its target. Our results further indicated that miR-338-5p was downregulated in placental tissues supporting larger twins of sIUGR, whereas epidermal growth factor-containing fibulin-like extracellular matrix protein 1 (EFEMP1) was upregulated. Moreover, miR-338-5p overexpression suppressed the growth and invasion of trophoblast cells. Importantly, results from luciferase reporter assay demonstrated that miR-338-5p bound on the 3′-UTR of EFEMP1. miR-338-5p suppressed the growth and invasion of trophoblast cells via targeting EFEMP1. Further, miR-338-5p/EFEMP1 might disrupt the function of trophoblast cells via inhibiting the phosphorylation of AKT.

**Electronic supplementary material:**

The online version of this article (10.1007/s43032-020-00160-3) contains supplementary material, which is available to authorized users.

## Introduction

Selective intrauterine growth restriction (sIUGR) is a serious complication of monochorionic (MC) diamniotic twins, which increases the risk of adverse pregnancy and poor postpartum outcomes [1]. sIUGR leads to severe growth restriction in the twin with the small placenta share and is considered a critical reason for intrauterine fetal demise (IUFD) and neurological dysfunction for both twins [2]. It has been confirmed that sIUGR is caused by inter-twin blood flow and unequal placental sharing [3, 4]. However, the underlying pathogenesis of sIUGR is still largely unclear.

MicroRNAs (miRNAs) are a group of 21–25-nucleotide-long non-coding RNA molecules, which play a multiple role in regulating cellular functions. miRNA commonly binds to the 3′-untranslated region of target gene and negatively mediates gene expression [5]. Moreover, miRNAs are indicated as potential biomarkers for pregnancy-specific diseases [6]. Further, our previous analysis has identified 14 specific differentially expressed miRNAs (DEMs) in larger twin placenta from pregnancies complicated with sIUGR than that in corresponding smaller twin placenta [7]. Therefore, gaining a deep insight into the molecular network of DEMs is a critical step for understanding the pathogenesis of sIUGR.

miR-338-5p, a brain-specific miRNA precursor, is involved in the tumorigenesis in certain human cancers [8, 9]. Moreover, miR-338-5p suppressed the growth and metastasis of glioblastoma cells through directly targeting EFEMP1 [10]. Importantly, our previous findings indicated that miR-338-5p is decreased in larger twins of sIUGR cases compared with that of smaller twins [7]. Further, trophoblast and stromal cells are the main cell types in human placenta, which are essential for pregnancy maintenance and fetal development [11]. Previous report has demonstrated that miR-128a contributes to preeclampsia through inducing the apoptosis of HTR-8/SVneo trophoblast cells [12]. Nevertheless, the biological function of miR-338-5p is less identified in trophoblastic cells.

Epidermal growth factor-containing fibulin-like extracellular matrix protein 1 (EFEMP1), also known as Fibulin-3, is broadly expressed throughout the body and essential in skeletal development [13]. Currently, EFEMP1 is widely recognized as an antagonist of angiogenesis, which presents dual role in tumorigenesis [14]. Previous report has indicated that EFEMP1 is an oncogene in glioma, while other findings elucidate the opposite effect of EFEMP1 in glioma [15, 16]. Moreover, EFEMP1 suppresses the metastasis of hepatocellular cancer cells via blocking the ERK1/2 activity [17]. Further, EFEMP1 is identified at E9.5 in the embryo proper and the expression lasts through embryogenesis until birth [18]. Furthermore, EFEMP1 is reported that highly expressed in placenta and located in basement membranes and at sites undergoing epithelial-mesenchymal transformation during embryogenesis [19]. However, the molecule network of EFEMP1 has not been fully explored in trophoblastic cells.

In the current study, we aimed to explore the function of miR-338-5p in trophoblastic cells and examined the connection between miR-338-5p and EFEMP1. Our findings demonstrated that miR-338-5p regulated trophoblast cells growth and invasion via targeting EFEMP1.

## Materials and Methods

### Tissues Specimens

A total of 10 women were enrolled in this research, including 5 cases complicated with sIUGR and others with normal MC. Placentas tissues around the individual insertion of umbilical cord were collected from monochorionic-diamniotic (MCDA) twin pregnancies after delivery. Then, the excised samples were washed in sterilized ice-cold saline (0.9% NaCl) for three times, stored at − 80 °C for following analyses. The intertwine EFW (estimated fetal weight) discordance was calculated as [(weight of the larger twin–weight of the smaller twin)/ weight of the larger twin]. The sIUGR group was defined by the EFW discordance greater than 25%. Normal MCDA twin pregnancies functioned as the control (without sLUGR or other complications). All pregnancies complicated with twin-to-twin transfusion syndrome (TTTs), severe congenital anomalies, intrauterine fetal death, chromosomal abnormalities, and maternal complications were excluded from present research. All patients were written informed consent. Our research was approved by the independent ethics committee of The Women’s Hospital, School of Medicine, Zhejiang University, China, and was in accordance with the Declaration of Helsinki.

### Cell Culture

Human HEK-293 T and trophoblast HTR-8/SVneo cells were obtained from the cell bank of Shanghai Biology Institute (Shanghai, China). Cells were seeded in RPMI1640 medium (Trueline, USA) that contains FBS (10%, 16,000–044, GIBCO, USA) and Penicillin-Streptomycin Solution (1%, P1400–100, Solarbio, China). All cells were incubated with the condition of 5% CO_2_ at 37 °C. This study was in agreement with the Declaration of Helsinki.

### Overexpression of miR-338-5p and EFEMP1

The hsa-miR-338-5p mimics (5’-AACAAUAUCCUGGUGCUGAGUG-3′) and microRNA negative control (5’-UUCUCCGAACGUGUCACGUUU-3′) were transfected into HTR-8/SVneo cells using Lipofectamine 3000 Transfection Reagent (L3000015, Thermo Fisher Scientific, USA). All procedures were guided according to the instruction in a previous reference [20].

For overexpression of EFEMP1, the full length of EFEMP1 cDNA was inserted into the lentiviral vector (pLVX-Puro). Then, the recombinant vector (pLVX-Puro-oeEFEMP1) was transfected into HEK-293 T cells. Meanwhile, the mock vector functioned as the corresponding negative control (oeNC).

### Quantitative Real-Time PCR (qRT-PCR)

Total RNA were extracted using the TRIzol Reagent kit (1596–026, Invitrogen, USA) and then converted into cDNA according to the instruction of the manufacturer (#K1622, Fermentas, Canada). Experiment was established on a real-time detection (ABI-7300, ABI, USA) using SYBR Green master mix (#K0223, Thermo, USA). Relative gene expression determination was counted according to the 2^-ΔΔCt^ method using β-actin as endogenous reference. Meanwhile, U6 RNA was used to normalize the expression of has-miR-338-5p (mature miRNA sequence: 5’-AACAAUAUCCUGGUGCUGAGUG-3′). Three replications were necessary for all reactions. The primers used in this research were listed in Supplementary File1**.**

### Western Blotting

Total protein was extracted using RIPA lysis buffer (JRDUN, Shanghai, China). The amount of 25 μg protein of each sample were fractionated via running on SDS-PAGE (10%) and subsequently transferred onto PVDF nitrocellulose membrane (HATF00010, Millipore, USA) for 12 h. Then, the membranes were probed with the primary antibodies overnight at 4 °C followed by the appropriate HRP-conjugated goat anti-rabbit IgG (A0208, Beyotime, China). Protein signals were analyzed using a chemiluminescence system. Relative protein expressions were normalized to β-actin. Each analysis was established in triplicate. Supplementary Table 1 provided details of primary antibodies.

### Cell Proliferation

In brief, cells transfected as indicated were planted in 96-well plates and cultured for 0, 12, 24, and 48 h. Cell proliferation was determined using Cell Counting Kit-8 (CCK-8) kits (CP002, SAB, USA). OD450 values of different cells were measured via using microplate reader (DNM-9602, Pulangxin, China). Three independently experiments were required for each time point.

### Cell Apoptosis

Briefly, cell apoptosis was determined by using commercial detection kit (C1063, Beyotime, China). All procedures were followed by the instruction in a previous reference [21]. Flow cytometer (Accuri C6, BD, USA) was utilized to determine cells at 48 h after infection. Three replications were needed for each sample.

### Cell Invasion

Transwell chamber assay with Matrigel-coated membranes (3422, COSTAR, USA) was utilized to examine cell invasion. Briefly, cells were suspended in medium containing 1% FBS and adjusted the concentration to 2 × 10^5^/mL, seeded in the upper chambers of 24-well Transwell plates (300 μL/well). After 24 h incubation, cells were fixed with formaldehyde (4%) and stained with crystal violet (0.5%). After that, the values for invasion were obtained by counting 5 random fields, magnification, 200 × .

### Luciferase Reporter Assay

In brief, the wild type (WT) and mutant 3′-UTR sequences of EFEMP1 were synthesized by Majorbio (Shanghai, China) and sub-cloned into pGL3 promoter vector containing the luciferase reporter. Both the wild type and mutant luciferase recombinant plasmids (1.5 ng) plus pRL-TK plasmid (20 ng) were co-transfected into the miNC or mimic cells respectively. A dual luciferase reporter assay (E1910, Promega, Madison, WI) was performed according to the manufacturer’s instructions at 48 h after transfection. The activity of fly luciferase was normalized to Renilla luciferase. Each analysis was repeated three times.

### Statistical Analysis

All data are expressed as means ± S.E.M and analyzed with GraphPad Prism software Version 7.0 (CA, USA). The results were assessed by analysis of variance (ANOVA). *P-*values < 0.05 were considered significant.

## Results

### miR-338-5p Is Upregulated in the Placental Tissue Supporting of the Smaller Fetus in sIUGR Pregnancies

**Fig. 1:**
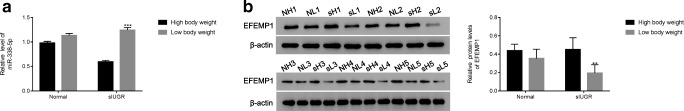
Differential expression of miR-338-5p and EFEMP1 in sIUGR twins. **a** miR-338-5p was upregulated in the placental share of the smaller fetus in sIUGR pregnancies, *** *p* < 0.001 vs high body weight. **b** The relative protein content of EFEMP1 was downregulated in the placental share of the smaller fetus in sIUGR pregnancies, ** *p* < 0.01 vs high body weight. NH: high body weight in normal twin, NL: low body weight in normal twin, sH: high body weight in sIUGR twin, sL: low body weight in sIUGR twin

Our preliminary findings indicated that miR-338-5p was increased in placenta tissues supporting smaller twins of sIUGR [7]. To further assess the level of miR-338-5p in human placental tissues, we compared miR-338-5p level between sIUGR group (sIUGR, *n* = 5) and normal group (normal, *n* = 5) using qRT-PCR. Clearly, the expression of miR-338-5p showed no significant difference between larger and smaller fetus in normal group. However, the level of miR-338-5p was much higher in the smaller fetus than that in larger fetus in sIUGR group (Fig. 1A).

Moreover, EFEMP1 is predicated as the target gene for miR-338-5p. Data was collected from Targetscan database (www.targetscan.org). Then, we examined the protein contents of EFEMP1 in sIUGR and normal group (*n* = 5) using western blot. As showed in Fig. 1B, the relative protein contents of EFEMP1 have no significant difference between larger and smaller fetus in normal group. Interestingly, the EFEMP1 content was downregulated in smaller fetus than that in larger fetus in sIUGR group.

### miR-338-5p Overexpression Suppressed the Growth and Invasion of Trophoblast Cells

**Fig. 2:**
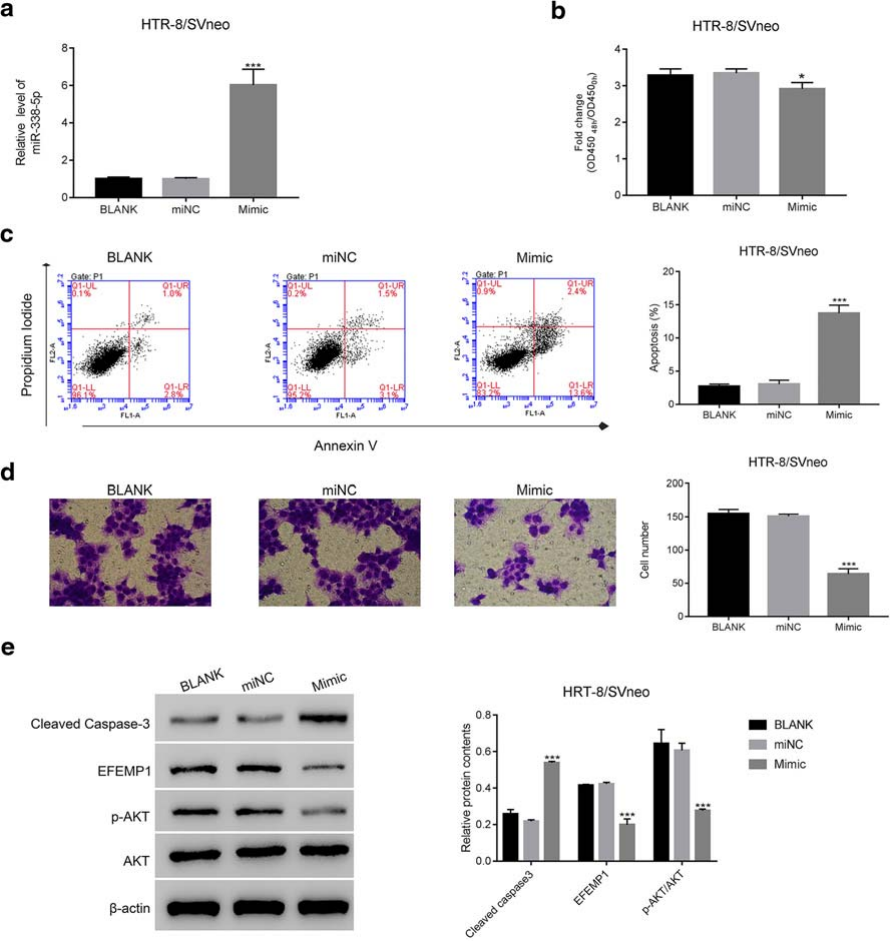
miR-338-5p overexpression suppressed the growth and invasion of trophoblast cells. **a** The level of miR-338-5p was significantly upregulated in mimic transfected cells, *** *p* < 0.01 vs miNC. **b** miR-338-5p overexpression inhibited the proliferation of trophoblast cells, * *p* < 0.05 vs miNC. **c** miR-338-5p mimic promoted the apoptosis of trophoblast cells, *** *p* < 0.001 vs miNC. **d** miR-338-5p overexpression suppressed the invasion of trophoblast cells, *** *p* < 0.001 vs miNC. **e** The protein contents of cleaved caspase-3, EFEMP1, and p-AKT/AKT were examined in blank, miNC, and mimic cells, *** *p* < 0.001 vs miNC

Trophoblast cells are the critical component for maintaining human placenta structure and function. To further address the function of miR-338-5p in human placenta, we induced gain of function assay by transfecting miR-338-5p mimic (Mimic) and corresponding negative control (miNC) into trophoblast cells, respectively. Clearly, miR-338-5p expression was significantly promoted in mimic transfected cells than that in miNC cells (Fig. 2A).

Next, CCK-8 assay was performed to determine the function of miR-338-5p in proliferation. Our results suggested that miR-338-5p overexpression inhibited the proliferation of trophoblast cells (Fig. 2B). Moreover, we found the apoptosis of mimic transfected cells was remarkably upregulated than that in miNC cells (Fig. 2C). Further, transwell assay indicated that the invasive ability of Mimic cells was deeply reduced compared with that in miNC transfected cells (Fig. 2D).

Caspase-3 is widely recognized as a pro-apoptosis factor [22]. Meanwhile, AKT signaling pathway is closely associated with cell growth [23, 24]. In the current study, we found the protein content of cleaved caspase-3 was upregulated in miR-338-5p mimic transfected cells. Moreover, overexpression of miR-338-50 deeply reduced the ratio of phosphorylation of AKT in total AKT (p-AKT/AKT) in trophoblast cells (Fig. 2E).

### miR-338-5p Targeted EFEMP1 in Human Embryonic Kidney Cells Through Binding on Its 3′-UTR

**Fig. 3:**
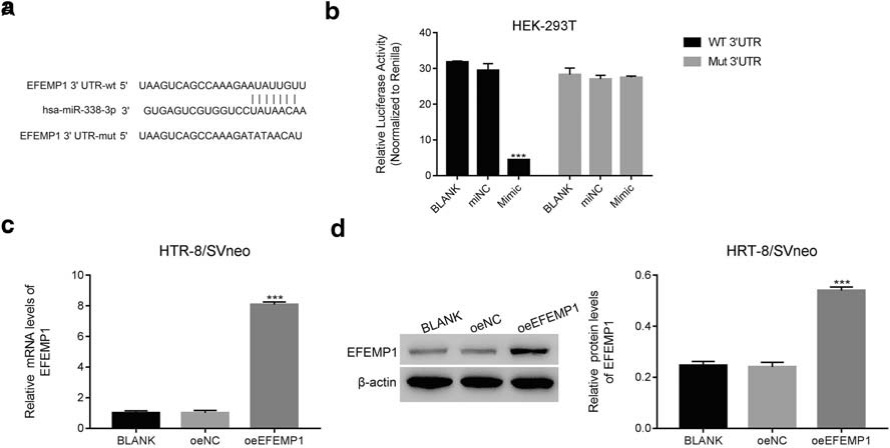
miR-338-5p targeted EFEMP1 in human embryonic kidney cells through binding on its 3′-UTR**. a** The putative miR-338-5p binding site in the 3′-untranslated region (3′-UTR) of EFEMP1 and the corresponding mutant sequence are presented as indicated. **b** The luciferase activity of the reporter driven by the wild type EFEMP1 3′-UTR was deeply suppressed in mimic cells as indicated above, *** *p <* 0.001 vs miNC. **c** and **d** The relative mRNA and protein levels of EFEMP1 were significantly upregulated in oeEFEMP1 transfected cells, *** *p <* 0.001 vs oeNC

Interestingly, the protein content of EFEMP1 was deeply decreased in mimic transfected cells (Fig. 2E). Then, the firefly luciferase report assay was utilized to further address the connection between miR-338-5p and EFEMP1. We synthesized luciferase report plasmids containing EFEMP1 3′-UTR sequence with the wild type (WT) or corresponding mutated miR-338-5p binding site (Mut) (Fig. 3A). Clearly, the relative luciferase activity of WT vector was deeply suppressed in mimic cells compared with that in miNC cells. However, the luciferase activity of Mut vector showed no significant difference between miNC and mimic cells (Fig. 3B).

### Overexpression of EFEMP1 in Trophoblast Cells

To further assess the function of EFEMP1, we induced EFEMP1 overexpression in trophoblast cells. The full length of EFEMP1 cDNA was inserted into pcDNA3.1(+) vector (oeEFEMP1).

Then, the recombinant vector was transfected into trophoblast cells. Meanwhile, the mock vector was functioned as corresponding negative control (oeNC). Clearly, both the relative mRNA and protein levels of EFEMP1 were significantly promoted in oeEFEMP1 transfected cells (Fig. 3C and D).

### EFEMP1 Overexpression Promoted the Growth and Invasion of Trophoblast Cells

**Fig. 4:**
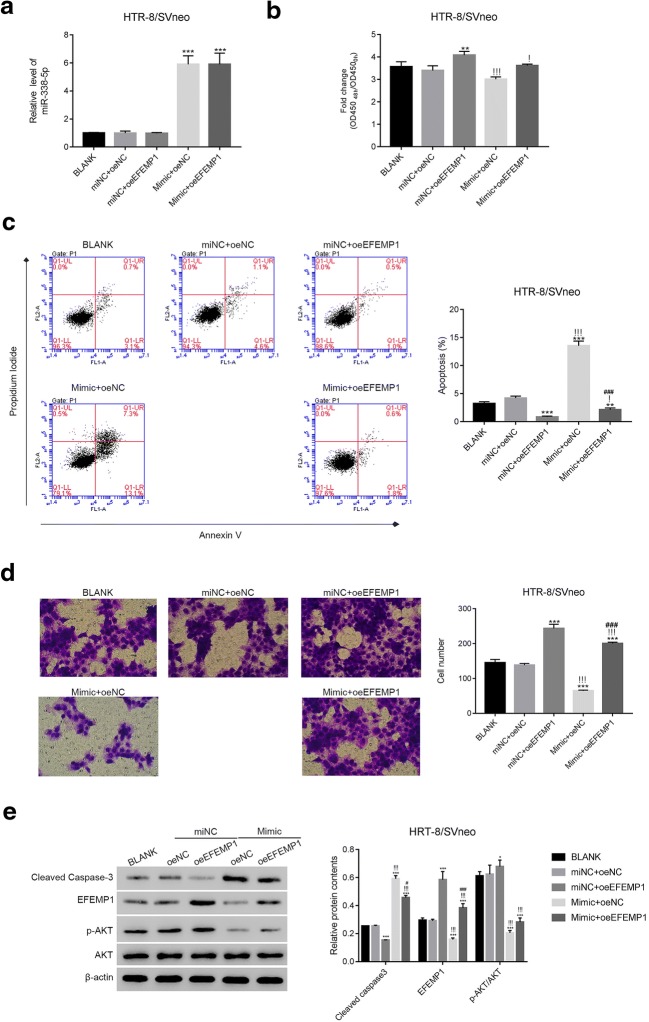
EFEMP1 overexpression promoted the growth and invasion of trophoblast cells. **a** The level of miR-338-5p was examined in different cells as indicated, *** *p* < 0.001 vs miNC. **b** EFEMP1 overexpression promoted the proliferation of trophoblast cells, ** *p* < 0.001 vs miNC+oeNC,! *p* < 0.05 vs miNC + oeEFEMP1,!!! *p* < 0.001 vs miNC + oeEFEMP1. **c** EFEMP1 overexpression inhibited the apoptosis of trophoblast cells; ** *p* < 0.01 vs miNC+oeNC; *** *p* < 0.001 vs miNC+oeNC;! *p* < 0.05 vs miNC+oeEFEMP1;!!! *p* < 0.001 vs miNC+oeEFEMP1; ### *p* < 0.001 vs mimic+oeEFEMP1. **d** EFEMP1 overexpression promoted the invasion of trophoblast cells; *** *p* < 0.001 vs miNC+oeNC;!!! *p* < 0.001 vs miNC + oeEFEMP1; ### *p* < 0.001 vs mimic + oeEFEMP1. **e** The relative protein contents of cleaved caspase-3, EFEMP1, and p-AKT/AKT were examined in different cells as indicated, * *p* < 0.05 vs miNC+oeNC, *** *p* < 0.001 vs miNC+oeNC,!!! *p* < 0.001 vs miNC + oeEFEMP1

Next, oeNC and oeEFEMP1 vectors were transfected into miNC or mimic transfected cells respectively. As presented in Fig. 4A, EFEMP1 overexpression did not affect the expression of miR-338-5p in trophoblast cells. Moreover, EFEMP1 overexpression significantly increased the proliferation of miNC cells, while this effect was decreased in mimic cells (Fig. 4B). Further, oeEFEMP1 deeply suppressed the apoptosis of miNC cells. Importantly, EFEMP1 overexpression also decreased the apoptosis of mimic cells (Fig. 4C).

Much importantly, our results indicated EFEMP1 overexpression promoted the invasion of trophoblast cells, whereas this effect was suppressed by the miR-338-5p mimic (Fig. 4D). We also found EFEMP1 overexpression significant inhibited the expression of cleaved caspase-3 in miNC or mimic cells, whereas increased the ratio of p-AKT/AKT (Fig. 4E).

## Discussion

sIUGR is a common disorder of twin pregnancies, which is caused with the condition of uneven placental sharing [25]. However, the pathogenies of discordant share of the placenta of MZ twins with sIUGR are far being fully understood. It has been indicated that miRNAs are associated with pregnancy-related diseases [26]. A recent report has identified that miR-210-3p overexpression impairs placentation of the smaller twin in sIUGR through inhibiting the expression of FGF1 [27]. Our previous report has indicated that miR-338-5p is upregulated in placenta tissues supporting smaller twins of sIUGR [7]. Currently, our results were consistent with our previous findings. Moreover, these analyses further indicated that miR-338-5p involved in the pathogenesis of sIUGR in placenta.

Trophoblast cells are the major component of the placenta. It has been confirmed that the invasiveness, differentiation and proliferation capabilities of trophoblasts are essential for implantation and placentation [28]. Importantly, growing evidences have demonstrated that miRNAs regulates the function of trophoblast cells [29–31]. In the current study, our findings firstly revealed that miR-338-5p overexpression blocked the growth of trophoblast cells. Moreover, the invasion ability of trophoblast cells was also downregulated in miR-338-5p mimic cells. Previous report has elucidated that inadequate invasion of trophoblast cells into the uterine arteries lead to defective placentation [32]. Hence, miR-338-5p might contribute to the progression of sIUGR via abolishing the function of trophoblast cells. However, there exist a difference between trophoblast cells and the primary trophoblast cells. Therefore, it will be valuable to further confirm these results in the primary trophoblast cells in the following study.

Previous report has indicated that miR-338-5p targets EFEMP1 in glioblastoma cells [10]. In this study, it was the first time to indicate that miR-338-5p also targeted EFEMP1 through binding on its 3′-UTR in trophoblast cells. Moreover, EFEMP1 presented pro-proliferation and anti-apoptosis role in trophoblast cells. Overexpression of EFEMP1 significantly improved the invasion of trophoblast cells. Further, EFEMP1 overexpression decreased the effects of miR-338-5p in trophoblast cells. Therefore, miR-338-5p might suppress the growth of trophoblast cells through targeting EFEMP1. Much importantly, our findings illustrated that potential value of miR-338-5p pathway in the prevention of sIUGR and indicated its signaling pathway in trophoblast cells.

Previous report has demonstrated that AKT pathway is involved in Nik-related kinase-mediated trophoblast proliferation and placental development [33]. Moreover, inhibiting the phosphorylation of AKT is associated with the metastasis of human trophoblast cells [34]. In the current study, we found the phosphorylation of AKT was positively correlated with the growth and invasion of trophoblast cells. Interestingly, EFEMP1 overexpression restored the phosphorylation of AKT in miR-338-5p mimic-transfected cells. Therefore, AKT might be a novel component in miR-338-5p/EFEMP1 pathway. Thus, miR-338-5p might disrupt the function of trophoblast cells via inhibiting the activity of EFEMP1/AKT pathway. Taken together, present research not only demonstrated the essential role of miR-338-5p in the development of placentation and fetal but also indicated its potential signaling pathway in trophoblast cells.

## Conclusion

In this research, we investigated the effect of miR-338-5p in trophoblast cells. Our findings elucidated that miR-338-5p suppressed the growth and invasion of trophoblast cells and targeted EFEMP1. This research further enhanced the understanding the role of miR-338-5p/EFEMP1 pathway in the development of placentation and fetal. It was highly valuable to confirm these results in primary trophoblast cells in the following studies.

## Electronic supplementary material

ESM 1 (JPG 124 kb)

ESM 2 (DOCX 18 kb)
