# Supplementary figures and images for: miR-338-5p Targets Epidermal Growth Factor-Containing Fibulin-Like Extracellular Matrix Protein 1 to Inhibit the Growth and Invasion of Trophoblast Cells in Selective Intrauterine Growth Restriction
Source: Reprod Sci. 2020 Feb 13;27(6):1357–64. doi: 10.1007/s43032-020-00160-3 (PMC7190678; doi:10.1007/s43032-020-00160-3)

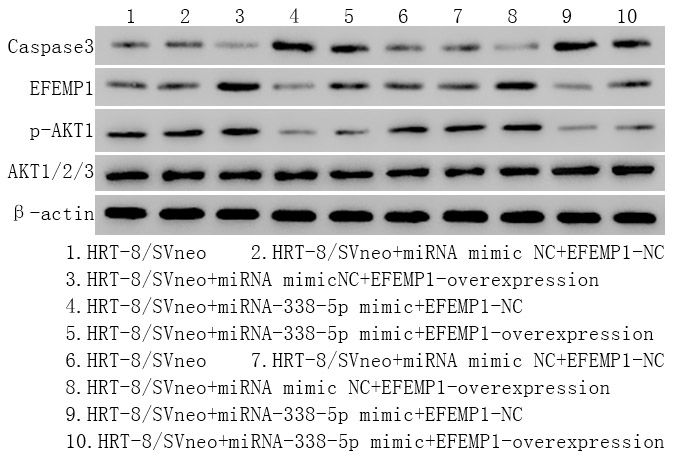

Supplement: Supplementary file 1 — (JPG 124 kb) [file 43032_2020_160_MOESM1_ESM.jpg]
