# Supplementary material for: miR-338-5p Targets Epidermal Growth Factor-Containing Fibulin-Like Extracellular Matrix Protein 1 to Inhibit the Growth and Invasion of Trophoblast Cells in Selective Intrauterine Growth Restriction
Source: Reprod Sci. 2020 Feb 13;27(6):1357–64. doi: 10.1007/s43032-020-00160-3 (PMC7190678; doi:10.1007/s43032-020-00160-3)
Supplement: Supplementary file 2 — (DOCX 18 kb) [file 43032_2020_160_MOESM2_ESM.docx]

**Supplementary File1: Primer sequence information**

**1.1 hsa-miR-338-5p MIMAT0004701**

RT-Primer：

5' GTCGTATCCAGTGCAGGGTCCGAGGTATTCGCACTGGATACGACCACTCA 3'

PCR Primer：

Primer F 5' CGCGAACAATATCCTGGTGC 3'

Primer R 5' AGTGCAGGGTCCGAGGTATT 3'

**1.2 Homo sapiens RNA, U6 small nuclear 1 (RNU6-1), small nuclear RNA**

NR_004394.1

Primer F 5’CTCGCTTCGGCAGCACA3’

Primer R 5’AACGCTTCACGAATTTGCGT3’

Pos: 4-97

Amplified product: Size: 94 bps

**1.3 Homo sapiens EGF containing fibulin extracellular matrix protein 1 (EFEMP1), transcript variant 2, mRNA**

**NM_001039348.3**

Primer F 5' TCTTCCAGATACAGGCCACAAC 3'

Primer R 5' CGAGCACAAGCATTGCACTTAC 3'

Pos: 1349- 1474

Amplified product: Size: 126 bps

**1.4 Homo sapiens beta actin; beta cytoskeletal actin, mRNA NM_001101**

Primer F 5' CATGTACGTTGCTATCCAGGC 3'

Primer R 5' CTCCTTAATGTCACGCACGAT 3'

Pos: 393-642

Amplified product: Size: 250 bps

**Supplementary Table 1: The primary antibodies information**

| Antibody name | Catalog | Source | Dilution factor |
| --- | --- | --- | --- |
| Cleaved Caspase3 | ab2302 | Abcam, UK | 1:500 |
| EFEMP1 | Ab106429 | Abcam, UK | 1:500 |
| p-AKT1 | Ab81283 | Abcam, UK | 1:2000 |
| AKT1/2/3 | Ab179463 | Abcam, UK | 1:10000 |
| β-actin | Ab8226 | Abcam, UK | 1:1000 |
